# Hypoxia inducible factor (HIF) as a model for studying inhibition of protein–protein interactions

**DOI:** 10.1039/c7sc00388a

**Published:** 2017-04-26

**Authors:** George M. Burslem, Hannah F. Kyle, Adam Nelson, Thomas A. Edwards, Andrew J. Wilson

**Affiliations:** a School of Chemistry , University of Leeds , Woodhouse Lane , Leeds LS2 9JT , UK . Email: a.j.wilson@leeds.ac.uk; b Astbury Centre for Structural Molecular Biology , University of Leeds , Woodhouse Lane , Leeds LS2 9JT , UK; c School of Molecular and Cellular Biology , Faculty of Biological Sciences , University of Leeds , Woodhouse Lane , Leeds LS2 9JT , UK

## Abstract

The modulation of protein–protein interactions (PPIs) represents a major challenge in modern chemical biology. Current approaches (*e.g.* high-throughput screening, computer aided ligand design) are recognised as having limitations in terms of identification of hit matter. Considerable success has been achieved in terms of developing new approaches to PPI modulator discovery using the p53/*h*DM2 and Bcl-2 family of PPIs. However these important targets in oncology might be considered as “low-hanging-fruit”. Hypoxia inducible factor (HIF) is an emerging, but not yet fully validated target for cancer chemotherapy. Its role is to regulate the hypoxic response and it does so through a plethora of protein–protein interactions of varying topology, topography and complexity: its modulation represents an attractive approach to prevent development of new vasculature by hypoxic tumours.

## Abstract 2

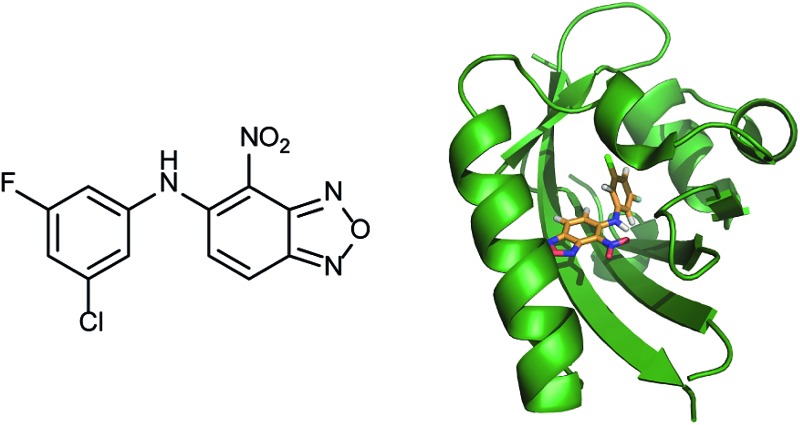
The state of the art in identifying protein–protein interaction inhibitors of hypoxia inducible factor – a promising target for anticancer drug design – is described.

## Introduction

As the proliferation of cancer cells is rapid, tumours quickly outgrow their blood supply resulting in a hypoxic environment. Hypoxia is an almost universal hallmark of solid tumours;^1^ the ability to adapt to hypoxic environments is crucial to their growth and survival^2,3^ and may therefore be exploitable in cancer therapy.^4^ In the main, this adaption is mediated by transcriptional activation of genes that facilitate short-term adaptive mechanisms (*e.g.* increased vascular permeability, vasodilatation, glucose transport, switch to anaerobic metabolism), as well as long-term adaptive mechanisms (*e.g.* angiogenesis).^5–8^ This co-ordinated homeostatic response is mediated in large part through the activation of the transcription factor hypoxia-inducible factor (HIF). HIF is responsible for activation/transcription of >100 genes which are required in order for cellular adaptation to hypoxia including oncogenes and inactivation of tumor suppressor genes. There are three isoforms of HIF: HIF-1, HIF-2 and HIF-3.^9^ Although the exact role of each isoform is not fully established, HIF-1 is considered to act as the primary messenger to activate transcriptional responses to hypoxia. HIF-1 is a promiscuous heterodimeric transcription factor; composed of an α subunit and a β subunit.^10^ HIF-1 activity in tumors is dependent upon the availability of the HIF-1α subunit, the levels of which increase under hypoxic conditions. The link between HIF-1 and cancer was established by immunohistochemical analysis of human cancer biopsies, with levels of HIF-1α increased in cancerous relative to normal tissue.^11^ Clinical data has also linked high levels of HIF-1α with resistance to some therapies, poor prognosis in malignancies and increased mortality.^12,13^ Experimental data has complemented clinical data, showing that in the absence of HIF-1α there is decreased tumour growth, vascularization and metastasis,^14^ whereas, the opposite prevails when HIF-1α is over expressed, thus highlighting a causal relationship between HIF-1α and cancer progression.^8,15,16^

**Fig. 1:**
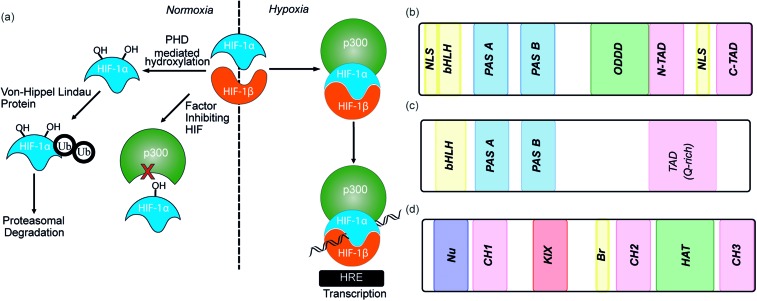
(a) Schematic depicting HIF pathway under normoxic and hypoxic conditions. HIF-1α – hypoxia inducible factor 1α, HIF-1β – hypoxia inducible factor 1β, PHD – prolyl hydroxylase domain, HRE – hypoxic response element (b) domain structure of HIF-1α. bHLH – basic helix-loop-helix, PAS – per ARNT-AHR-Sim, ODDD – oxygen-dependent degradation domain, NTAD – N-terminal transactivation domain, CTAD – C-terminal transactivation domain (c) domain structures of HIF-1β (d) domain structure of p300/CBP.

The β subunit of HIF-1 (sometimes known as aryl hydrocarbon receptor nuclear translocator, ARNT) is constitutively expressed in the nucleus whereas the stability, subcellular localization and transcriptional potency of the α subunit is regulated by oxygen dependent post-translational modifications and therefore oxygen concentration.^4^ HIF-1α is continuously expressed at a low level in the cell, but under normoxic conditions is rapidly degraded, most prominently by the hydroxylation of two proline residues by oxygen reliant HIF-prolyl hydroxylases (PHDs).^2,17,18^ resulting in binding of von Hippel–Lindau tumor suppressor (pVHL), the recruitment of an E3 ligase complex and ubiquitin-mediated proteasomal degradation (Fig. 1a).^9^ Due to the rapid nature of this process HIF-1α has a half-life of less than 5 minutes under normoxic conditions, resulting in no detectable protein in normoxic cells.^19^ Although most prominent, the pVHL pathway is not the only pathway controlling levels of HIF-1α. A further mechanism for HIF-1α regulation is through recruitment of the human double minute 2 (*h*DM2) ubiquitin-protein ligase resulting in interaction with the tumor suppressor p53 and ultimately proteasomal degradation.^20^ In addition, Hsp90 interacts directly with HIF-1α and has been suggested to promote a conformational change in HIF-1α, which leads to inhibition of the dimerization with HIF-1β.^21^

Under hypoxic conditions there is a decreased rate of HIF-1α degradation; it accumulates, translocates to the nucleus and forms a heterodimer with HIF-1β where it engages in PPIs with transcriptional co-activators, such as the CH1 domain of p300.^22,23^ The HIF-1α/p300 interaction is abrogated under normoxic conditions in an additional oxygen-dependent process through hydroxylation of Asn803, located within the C-TAD of HIF-1α. Hydroxylation of Asn803 is mediated by an asparaginyl hydroxylase known as factor inhibiting HIF-1 (FIH-1), preventing interaction of HIF-1α with the CH1 domain of p300 (Fig. 1a).^24^ The HIF-1 dimer/p300 complex binds to hypoxic response elements (HRE) on DNA and causes a plethora of downstream events *via* transcription mediation (Fig. 1a).^25^ Hypoxic response elements have many roles in normal and cancer biology including: the promotion of angiogenesis,^26^ stem cell maintenance,^27^ metabolic reprogramming,^28^ autocrine growth factor signalling,^29^ metastasis^30^ and providing a mechanism of resistance to radiation and chemotherapy.^31^ It is thus unsurprising that there are many potential molecular mechanisms to inhibit HIF activity, including decreasing mRNA levels, decreasing protein synthesis, increasing degradation, inhibiting protein–protein interactions (PPIs) of HIF, inhibiting the HIF/DNA interaction and decreasing the transcriptional activity of HIF.^31^ The near universality of hypoxia in human tumors and the centrality of the non-redundant HIF pathway in adapting to the hypoxic environment suggest that inhibition of the HIF pathway could reduce angiogenesis thereby contributing directly to tumour cell death^32^ and may have therapeutic antitumor utility.

This review will outline efforts to develop inhibitors of HIF function with an emphasis on targeting the numerous protein–protein interactions of the HIF transcription factor. Consequently the article begins with an overview of HIF structure. For clarity, a brief overview of indirect methods to target HIF function is given, before a more extended discussion of the various approaches taken to develop inhibitors of HIF protein–protein interactions. The majority of the review focusses on HIF-1, however a number of highly significant recent articles on HIF-2 are included to highlight the power of various different ligand discovery approaches in modulating HIF biology.

## Structural biology of the HIF family

The number of HIF structures has significantly increased in recent years allowing the structural biology of HIF to be explored (Table 1).

**Table 1:** Summary of the currently available HIF structures

Structure	Structure detail	PDB ID	Ref.
HIF dimers	HIF-1α/ARNT	4H6J	33
	HIF-2α/ARNT/co-activator complex	4PKY	34
	HIF-2α/ARNT complex	3F1P	35
	HIF-2α/ARNT complex with an artificial ligand bound	3F10	35
	HIF-2α/ARNT complex with a benzoxadiazole ligand bound	4GS9, ; 4GHI	36 and 37
	HIF-2α–ARNT bound to PT2399	5UFP	38
	HIF-2α–ARNT bound to PT2385	5TBM	39
	HIF-2α–ARNT PAS domain bound to tetrazole containing antagonist	4ZPK	40
	HIF-2α–ARNT complex bound to proflavin	4ZPH	40
	HIF-2α–ARNT complex with HRE DNA	4ZPR	40
	HIF-2α–ARNT bound to benzoxadiazole antagonist	4ZQD	40
	HIF-2α–ARNT bound to THS017	3H7W, ; 3H82	41
	HIF-2α–ARNT complex with ethylene glycol	3F1N	35
HIF–FIH complexes	FIH in complex with HIF-1α	1H2K, ; 1H2L, ; 1H2M	42
	FIH (D201E) complex with HIF-1α and α-ketoglutarate	5JWP	43
		3D8C, ; 2ILM	44
HIF–PHD complexes	PHD2 in complex with 2OG and HIF-1α CODD	5L9B, ; 5L9V, ; 5LA9, ; 5LAS	45
	PHD2 in complex with NOG and HIF-1α	3HQR	133
vHL–HIF complexes	vHL/elongin/B-elongin/C-elongin complex bound to HIF-1α	4AJY	46
		1LQB	47
		1LM8	48

HIF-1 is a heterodimer consisting of two subunits; an oxygen-sensitive HIF-1α subunit and a constitutively expressed HIF-1β subunit, both subunits are members of the basic helix-loop-helix (bHLH) proteins of the PER-ARNT-single-minded protein (SIM) (PAS) family of transcription factors (Fig. 1b).^49^ The regulation of HIF-1α is dependent on the oxygen dependent degradation domain (ODDD – the region upon which PHDs act) and two transactivation domains: the N-terminal transactivation domain or N-TAD and the C-terminal transactivation domain or C-TAD (Fig. 1b, for domain structure of HIF-1β see Fig. 1c).^50^ The C-TAD is involved in modulating the transcriptional activation of HIF-1α under hypoxic conditions, in contrast to the N-TAD, which is involved in the stabilisation of HIF-1α. The N terminal region of HIF has a basic helix-loop-helix (HLH) domain and enables binding of HIF to the hypoxia response elements (HRE).

**Fig. 2:**
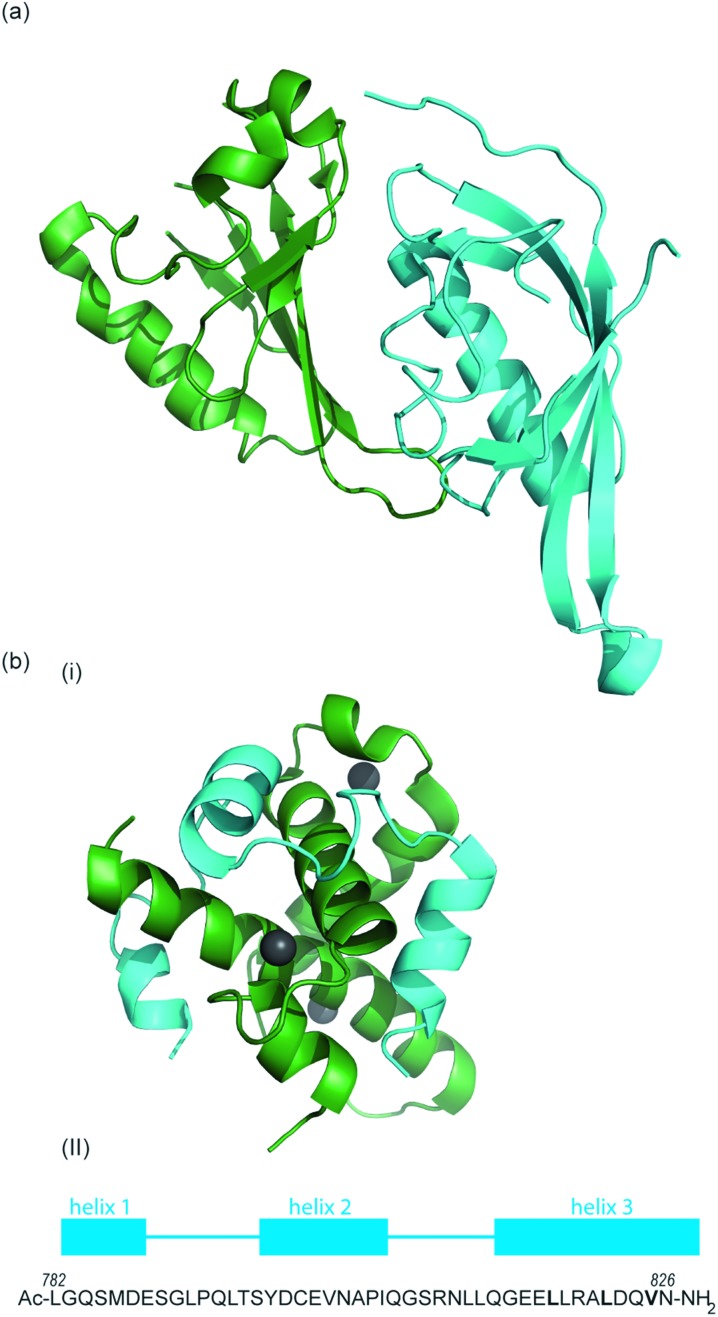
(a) Structure of the heterodimeric HIF-1α: ARNT PAS-B complex excised from its ternary complex with DNA (one subunit cyan, one forest green) PDB ID: 4ZPR (b) (i) NMR structure of complex between HIF-1α CTAD (cyan) and p300 CH1 domain (forest green) PDB ID: ; 1L8C (ii) primary sequence of HIF-1α CTAD with helical regions indicated.

As previously discussed, HIF-1 is a heterodimer of HIF-1α and HIF-1β (aka ARNT) but there are 2 other α isoforms, known as HIF-2α and HIF-3α. Whilst both can form dimers with HIF-1β, HIF-3α lacks the ability to bind the co-activator protein and thus is inactive.^51^ When HIF-2α and HIF-3α form complexes with HIF-1β they are known as HIF-2 and HIF-3 respectively and have been reported to be expressed in different amounts in different tissues.^52,53^ Dimerization occurs through a bHLH domain and 2 per-ARNT-AHR-Sim (PAS) domains on both the HIF-1α subunit and the HIF-1β subunit (*e.g.* Fig. 2a).^54,55^ PAS domains are implicated in protein–protein interactions in other systems and adopt a range of diverse homo/heterodimerization binding modes.^56^ It is also thought that coiled coil co-activators play a role in HIF α/β dimerization.

The co-activator protein p300/CBP is thought to control gene expression by relaxation of the chromatin structure at the gene promoter *via* intrinsic histone acetyltransferase activity; it also recruits basal transcriptional machinery including RNA polymerase to the promoter.^57^ The multidomain proteins p300 and CBP are very similar in structure, they consist of key domains (Fig. 1d) including; the nuclear interaction domain (Nu), the CREB and MYB interaction domain (KIX), cysteine/histidine regions (CH/TAZ), a histone acetyltransferase domain (HAT) and a bromodomain (Br).^58^ The CH1 domain (sometimes known as transcriptional adapter zinc-binding (TAZ)1 domain of p300)^59^ of each protein interacts with the CTAD of HIF-1α. The CH1 domain is also the binding site for the CREB-binding protein/p300-interacting transactivator with ED-rich tail (CITED) family of proteins which can compete with HIF-1α.^60,61^ In this review the majority of the discussion centres upon the CH1 domain which for p300 and CBP differs by only 5 amino acids; for clarity we will refer only to p300.

To date, no X-ray crystal structure of the HIF-1α/p300 complex has been reported. The interaction between the CH1 domain of p300 and the C-TAD of HIF-1α was solved by using multidimensional NMR methods (Fig. 2b, PDB: ; 1L8C, ; 1L3E).^62,63^ The p300 CH1 domain forms a rigid structure consisting of 4 helices constrained and stabilised by binding 3 zinc atoms. The HIF-1α CTAD is thought to be unstructured in the absence of p300; upon binding, HIF-1α forms three helical regions which mediate the interaction between the two proteins. Biochemical and biophysical studies have highlighted key regions of HIF-1α (helix 2 and helix 3)^64,65^ that interact with the CH1 domain of p300 by hydrophobic or polar interactions. Mutational studies have indicated the key binding residues of HIF-1α. Cys_800_ ^66,67^ and Asn_803_ (a substrate for FIH discussed earlier)^24,62^ located in helix 2 have been highlighted as key binding residues in a range of assays, however these two residues are polar which is unusual for PPI hotspots. Helix 3 contains 3 key hydrophobic binding residues, Leu_818_, Leu_822_ and Val_825_.^67,68^ Asp_823_ and Gln_824_ have also been suggested to represent important residues.^69,70^ On p300 Leu-344, Leu-345 have been identified as important residues^67^ whilst a separate study identified His_20_, Leu_47_, Ile_71_ as being important with the last of these the most significant.^65^

In addition to p300 which recognises HIF-1α, co-activators can also bind to the other subunit of the HIF complex *i.e.* HIF-1β/ARNT; HIF complexes utilize several co-activator proteins including thyroid hormone receptor interacting protein 230 (TRIP230),^71^ coiled-coil coactivator (CoCoA),^72^ and transforming acidic coiled-coil 3 (TACC3)^73^ at different promoters. Whereas inhibition of HIF-1α/p300 represents a specific means to attenuate HIF-1α function, the fact that HIF-1β is constitutively expressed renders the inhibition of HIF-1β/coiled-coil coactivator interactions amenable to modulation of all HIF complexes.

There are many different pathways for HIF stabilisation and increased activity in hypoxic environments. HIF is involved in many signalling pathways meaning there are multiple potential targets for small molecule intervention (available structures are summarised in Table 1). HIF inhibitors can be broadly classified by their mechanism of action. Although, one common denominator of most, if not all, HIF inhibitors identified until recently is a lack of specificity, indicative of hitting multiple targets and pathways; HIF inhibition cannot be easily separated from other activities exerted by these agents. This means that mechanism of action can be difficult to decipher and is compounded by the fact that many known inhibitors were discovered through cell-based screening, which offers little information regarding the mechanism of action. The five means by which HIF can be modulated are: HIF mRNA expression, HIF protein translation, HIF protein degradation, HIF DNA binding and HIF transcriptional activity.

## Overview of indirect HIF modulation

### HIF-1α mRNA expression

It has been suggested that, under hypoxic conditions, levels of HIF-1α mRNA may be a limiting factor affecting the rate of HIF-1α protein translation.^74^ Molecule EZN-2698 is an RNA modulator, which is composed of a third-generation oligonucleotide; a technology that specifically binds and inhibits the expression of HIF-1α mRNA. It has shown potent (IC_50_ = 1–5 nM) and selective inhibition of HIF-1α mRNA and protein expression in both normoxia and hypoxia. Mice models demonstrated dose-dependent and highly potent down regulation of endogenous HIF-1α and VEGF in the liver. Tumor reduction was found in nude mice implanted with DU-145 human prostate cancer cells treated with EZN-2968.^75^ This indicated inhibition of HIF-1α mRNA has potential as a target for cancer therapy.

### HIF-1α protein translation

**Fig. 3:**
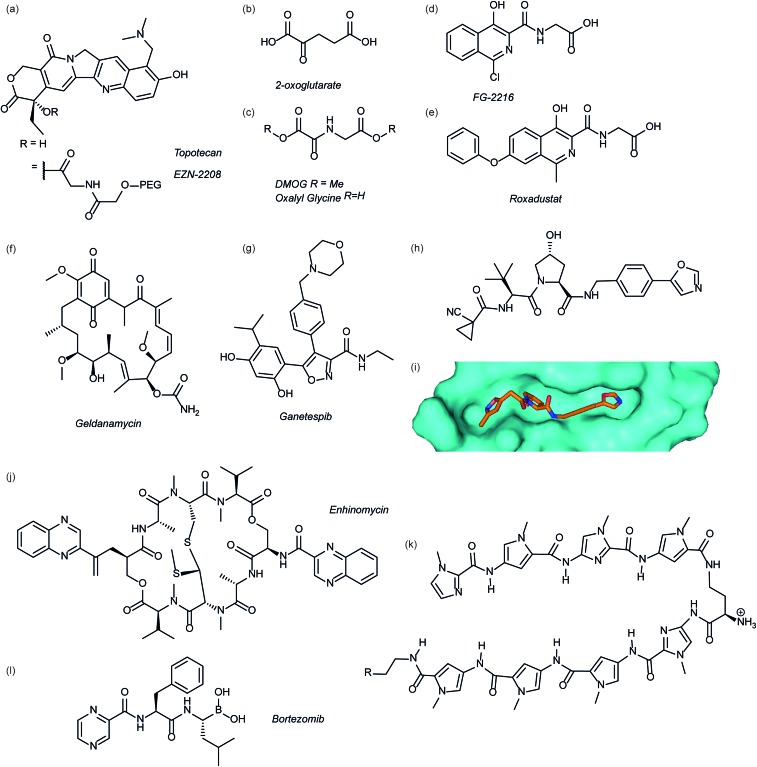
Modulators of the HIF pathway (a) topotecan and derivative EZN-2208 (b) 2-oxoglutarate (c) DMOG and oxalyl glycine (d) FG-2216 (e) roxadustat (f) geldanamycin (g) ganetespib (h) X-ray crystal structure of a hydroxyproline derived inhibitor (orange) bound to pVHL (cyan), PDB ID: 3zrc (i) optimised hydroxyproline derived pVHL inhibitor VH298 (j) echinomycin (k) DNA sequence specific polyamide (l) bortezomib.

Several agents have been described that may affect the rate of HIF-1α protein synthesis. One such agent is topotecan (Fig. 3a), an FDA approved drug currently used as a second line therapy for patients with small cell lung or ovarian cancer. Topotecan works by inhibiting topoisomerase I, ultimately abrogating HIF-1α translation.^76^ Recently, it has been shown that administration of daily topotecan in combination with the anti-VEGF antibody bevacizumab exerts synergistic antitumour activity in xenograft models, providing a rationale for clinical development of this combination strategy.^77^ Other topoisomerase 1 inhibitors have been developed, including EZN-2208 (Fig. 3a).^78^ EZN-2208 has better pharmacokinetic properties and a longer half-life than topotecan, making it more suitable for chronic suppression of the HIF-1 pathway. Other agents and targets that affect HIF-1α protein translation include; digoxin, a cardiac glycoside, which inhibits the translation of HIF-1α by an mTOR-independent mechanism,^79^ and PX-478, an agent that potentially inhibits HIF-1α translation through multiple mechanisms, although none have been confirmed.^80^

### HIF-1α degradation pathway

Since the oxygen dependent hydroxylation of HIF-1α is required for its degradation, inhibition of the PHD enzymes responsible for said hydroxylation provides an attractive target for therapeutic intervention. Several inhibitors of PHD2 have been reported and entered clinical trials, this area has recently been reviewed elsewhere and so will not be covered in detail here.^81^ Perhaps the most commonly used PHD2 inhibitor in cell culture experiments is dimethyloxalyl glycine (DMOG) which acts as a prodrug for *N*-oxalylglycine (Fig. 3c).^82^ *N*-Oxalylglycine acts as a competitive inhibitor of the co-factor oxoglutarate (Fig. 3b) which is crucial for PHD enzymatic turnover.^83^ Whilst DMOG is a useful research tool and serves as a proof of principle for PHD inhibition it also inhibits many other 2OG oxygenases. Early derivatives of oxalyl glycine were able to yield some selectivity between PHD2 and FIH.^84^ Subsequently medicinal chemistry and structural biology efforts have yielded a range of clinical candidates including FG-2216 (ref. 85) and Roxadustat (Fig. 3d and e).^86^ Whilst many of these compounds are 2-oxoglutarate co-factor competitors, as the structural details are elucidated the development of HIF competitive PPI inhibitors may provide an exciting avenue of research.^45^

Heat shock protein 90 (Hsp90) is a molecular chaperone that controls the folding and regulates the function of many proteins, including receptor tyrosine kinases, serine/threonine kinases, transcription factors and activated oncoproteins.^87^ Disruption of Hsp90 function has been shown to promote HIF-1α degradation *via* a novel, oxygen-independent E3 ubiquitin ligase and diminishes HIF-1α transcriptional activity.^88^ HIF-1α heterodimers may also not acquire the proper conformation and therefore fail to recruit cofactors important for HIF-1-mediated transcriptional activity.^89^ The first Hsp90 inhibitor was the natural product, geldanamycin (Fig. 3e),^88^ which exerted its inhibitory activity by competing with the ATP binding site. Another Hsp90 inhibitor, ganetespib (Fig. 3f), with enhanced drug-likeness compared to geldanamycin, has been shown to induce HIF-1α degradation *in vivo* in a triple-negative breast cancer model^90^ and is currently in phase III clinical trials (ClinicalTrials.gov Identifier: NCT01798485).

Considerable efforts have been made to identify inhibitors of the protein–protein interaction between HIF-1α and pVHL. Using fragment based approaches a hydroxyproline based fragment was identified that could be grown into μM inhibitors.^91,92^ Crystal structures of these ligands (Fig. 3h)^91,93^ have enabled structure based improvement to yield ligands with nM affinity for pVHL and more recently cell-permeable analogues that represent ideal chemical probes (Fig. 3i).^94^ pVHL ligands have also proven useful during the development of PROTACs.^95^

### HIF-1 binding to DNA

Inhibition of HIF-1 DNA binding to the hypoxia responsive element (HRE); a step required for transcription induction, is also a potential mechanism by which small molecules may inhibit HIF-1 activity. Proof of principle for this approach has been established using a cyclic peptide, echinomycin (Fig. 3j), which was known to bind DNA in a sequence-specific fashion.^96^ It was shown that echinomycin inhibits the DNA/HIF-1 interaction more potently than DNA/AP-1 or DNA/NF-κB, binding, providing evidence of selective inhibition based on recognition of DNA sequences. Dervan type polyamides,^97,98^ which have a similar mechanism, have also been developed to modulate HIF/DNA interactions (Fig. 3k).^97^

### HIF-1α transcriptional activity

Whilst inhibition of the proteasome leads to an accumulation of HIF-1α, the HIF-1α that accumulates is transcriptionally inactive.^99^ A proteasome inhibitor, bortezomib (Fig. 3l), has been FDA approved for treatment of numerous cancers. In addition to its role in proteasome inhibition bortezomib was shown to limit the HIF-1α/p300 interaction, by improving the binding of FIH to HIF-1α.^100^

## Small molecule inhibitors of HIF PPIs

**Fig. 4:**
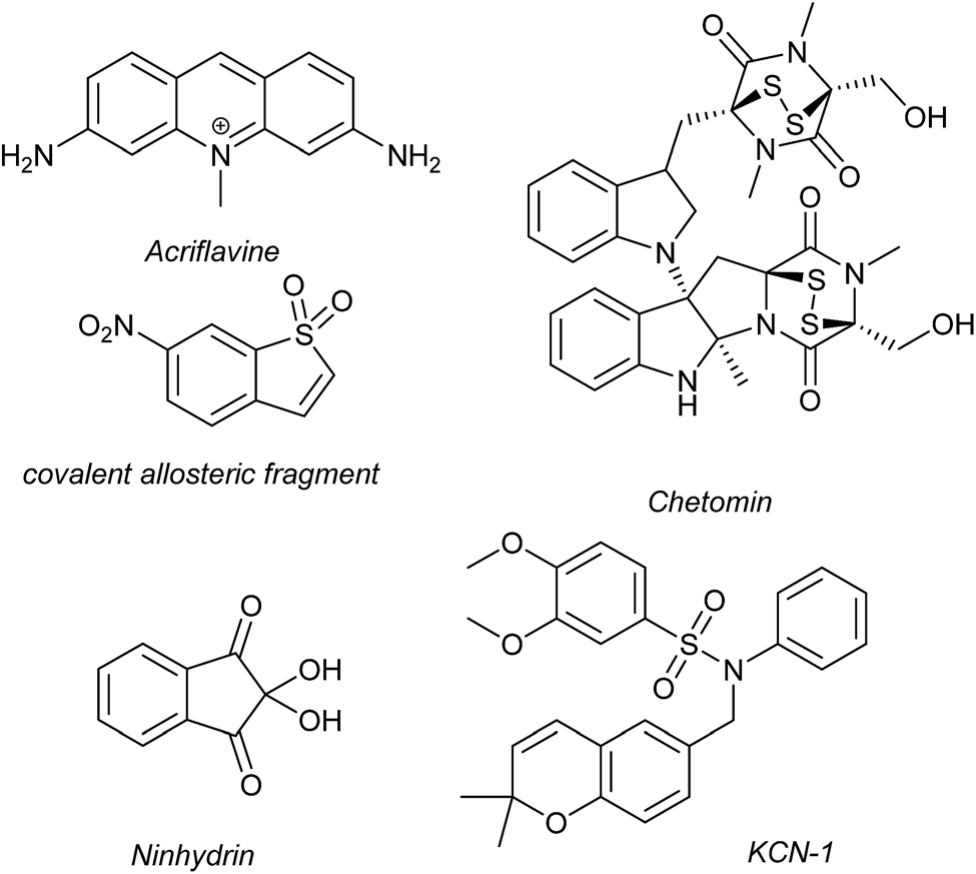
Reported small molecule HIF-1α/p300 inhibitors.

Several families of compounds have been identified which inhibit the interaction between HIF-1α and HIF-1β. Acriflavine (Fig. 4) was identified as an inhibitor of dimerization in a screen of compounds that had previously entered phase II clinical trials.^101^ A covalent fragment screening approach also identified an allosteric small molecule inhibitor of the HIF-1α/HIF-1β PPI.^33^

There have been efforts to directly disrupt the HIF-1α/p300 interaction, thus far with limited success. A natural product, chetomin, was identified as an inhibitor and shown to be effective at down-regulating HIF-controlled transcription as well as reducing tumour size in mouse models.^32^ The epidithiodiketopiperazine (ETP) functionality contained in chetomin (Fig. 4) was subsequently shown by the Schofield group to disrupt folding of the CH-1 domain of p300, *via* zinc ejection, preventing interaction with HIF-1α.^102^ Indeed, much simpler ETP containing compounds have been shown to be sufficient for activity.^103^ Additional families of compounds capable of disrupting the folding of the p300 CH1 domain by zinc ejection have been identified, including ninhydrin (Fig. 4).^104^ Compounds with zinc ejection-based mechanisms are likely to encounter issues with selectivity and toxicity, due to interactions with other zinc-binding proteins and the fact that p300 has multiple binding partners, so are unlikely to represent viable therapeutics.^102^ Care should be taken in future screening campaigns to exclude metal binding moieties or at the very least confirm that any hits are not acting *via* this mechanism.

A small molecule called KCN-1, reported to inhibit the HIF-1α/p300 interaction, was identified through high-throughput cell-based screening of a combinatorial library,^105^ with several SAR studies carried out in follow-up.^106^ KCN-1 has been shown to prevent HIF-regulated expression and reduce tumour size in animal models but the exact mode of action still remains unclear.^107^ In our hands, there was no evidence that this molecule inhibited the HIF-1α/p300 interaction in a fluorescence anisotropy competition assay.^108^

**Fig. 5:**
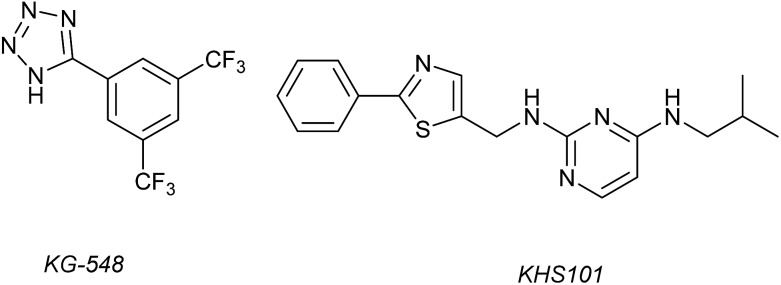
Small molecule modulators of ARNT/TACC3 interaction.

^1^H–^15^N HSQC complexation-induced shifts were used to identify inhibitors of the ARNT/TACC3 interaction focusing on fragments that recognise the PAS-B domain (see Fig. 1c for domain structure of HIF-1β).^109^ Of the 760 compounds tested, a number inhibited ARNT/TACC3 and gave interesting results in terms of molecular mode of action. Compound KG548 (Fig. 5) was shown to bind to a cavity on ARNT-PAS-B proximal to the TACC3 binding site resulting in competitive inhibition. Selectivity over ARNT2, BMAL-1 and HIF-2α was observed. Alpha screen and immunoprecipitation experiments in lysates of HEK 293T cells indicated the compounds could act as a competitive inhibitor of the protein–protein interaction in a dose dependent manner, albeit with limited potency (IC_50_ ∼25 μM). From the same screening workflow, the authors identified a further compound KHS101 (Fig. 5); *in vitro* pull down and ^1^H–^15^N HSQC were indicative of the compound not binding directly to ARNT-PAS-B or acting as a competitive inhibitor. Pulse chase experiments using cycloheximide (CHX) as a translation inhibitor established that KHS101 acts to destabilize TACC3.

**Fig. 6:**
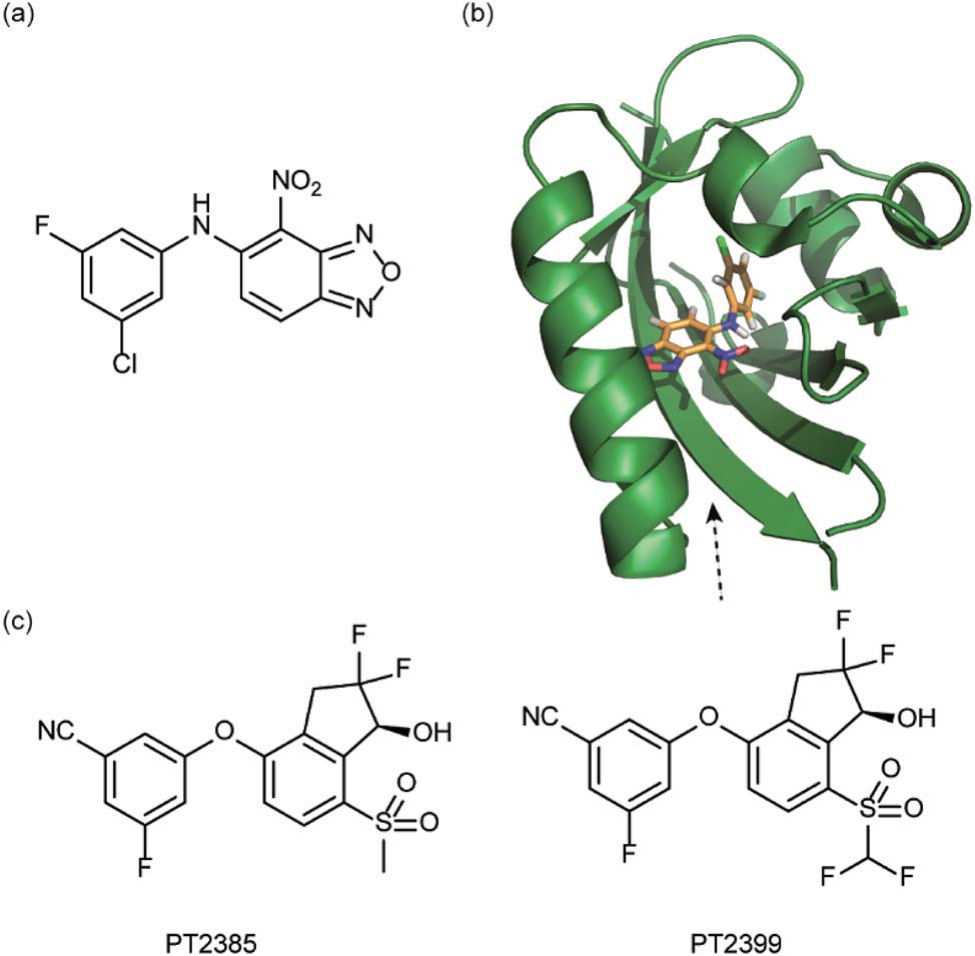
HIF-2 heterodimerization inhibitors (a) structure of a HIF-2α-PAS B ligand able to inhibit dimerization with ARNT PAS B through an allosteric mechanism (b) structure (PDB ID 4GHI) of compound shown in (a) (orange) bound to HIF-2α-PAS B (green), the arrow points to the β-sheet distal to the site of small molecule binding where HSQC shifts were observed and used to rationalise inhibition of heterdimerization with ARNT (c) compounds used to validate HIF-2 as a target in renal cancers.

In a related manner, the PAS-B domain from HIF-2α has been shown to be amenable to small molecule binding and consequently allosteric regulation. The PAS domain contains a relatively large (290 Å^3^) preformed cavity that can bind ligands, identified using solution NMR-based screening of a fragment library (∼800 compounds);^35^ however the hit compounds identified exhibited only modest inhibition of PAS–PAS interactions. A high throughput *in vitro* screen was thus developed that allowed screening of >200 000 compounds and resulted in the identification of 70 candidate HIF-2α/ARNT inhibitors which following optimisation resulted in a compound (Fig. 6a) with HIF-2α-PAS B affinity of *K*_d_ = 81 nM.^37^ X-ray (Fig. 6b) and NMR studies have established that binding of this ligand to the PAS-B domain results in conformational changes to the PAS-B domain that changes the ARNT binding β-sheet surface of the HIF-2α PAS-B domain. The ligand has been shown to selectively disrupt the HIF-2 heterodimerization selectively over HIF-1 and inhibit HIF-2 assembly in cells, retarding DNA-binding activity and reducing HIF-2 target gene expression. Medicinal chemistry efforts have developed understanding of the SAR surrounding this compound class.^36,40^ Subsequently this inhibitor class (Fig. 6c) has been used to validate HIF-2 as a viable cancer target in renal cell cancer models.^38,39,110^

## Identification of hits against HIF PPIs using biological selection methods

**Fig. 7:**
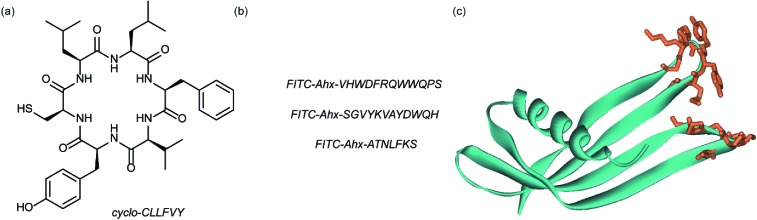
Structures of peptide and protein derived modulators of HIF PPIs identified using selection methods (a) cyclic peptide inhibitor of HIF-1α/HIF-1β dimerization identified using SICLOPPS (b) FITC functionalized p300 binding peptides identified by phage display (c) X-ray structure of p300 binding Affimer (cyan with binding loops in orange) PDB ID: 5A0O.

Dimerization of HIF-1α and HIF-1β is critical for both transcriptional activity and DNA binding and therefore has been described as an optimal point of interception. The Tavassoli group used a genetically encoded HTS platform for the identification of cyclic peptides that are able to disrupt the dimerization. Using a HIF-1 bacterial reverse two-hybrid system and a plasmid-encoded split intein circular ligation of peptides and proteins (SICLOPPS)^111^ library of 3.2 million cyclic hexapeptides, a cyclic peptide – cyclo-CLLFVY – was identified (Fig. 7a).^112^ The compound was tested *in vitro* and in cells (using a luciferase reporter assay) and was shown to disrupt HIF-1 dimerization by binding the PAS-B domain of HIF-1α. With a *K*_d_ of 124 (±23) nM. The compound was shown to be capable of inhibition of HIF-1α/HIF-1β in MCF-7 and U2OS cells as evidenced by a proximity ligation assay and resulted in a reduction in hypoxia mediated VEGF expression. No evidence for inhibition of HIF-2 was observed. The conditional expression of cyclo-CLLFVY in a human cell line has recently been reported.^113^

Our group used phage displayed Affimers to identify peptide-based inhibitors of HIF-1α/p300.^65^ Using biotin-tagged p300 (obtained through sortase-mediated N-terminal labelling).^114^ A N.E.B.^115–117^ phage library was used to identify binders with next generation sequencing using the Illumina platform,^118^ demonstrating enrichment over three panning rounds. Three peptides (Fig. 7b) were selected for synthesis: VHWDFRQWWQPS, SGVYKVAYDWQH and ATNLFKS, each of which was labelled with fluorescein and tested for interaction with p300 in a fluorescence anisotropy assay; the highest affinity peptide was VHWDFRQWWQPS with an affinity of 20.67 (±3.17) μM. ^1^H–^15^N HSQC experiments were performed to locate the binding site of the phage display-derived peptide on p300 – these indicated that VHWDFRQWWQPS may bind towards the top of the helix 3 binding pocket. A reduction in binding affinity for the p300 variants L47M and I71M corroborated this observation. The second phage display experiment used non-antibody binding proteins presented on the surface of the phage. These Affimers are derived from a phytostatin consensus sequence and exhibit enhanced properties for biotechnology *e.g.* soluble and easy to express in *E. coli*.^119^ The Affimer scaffold has two randomised loops of 9 residues for recognising protein targets (Fig. 7c). Following panning, three Affimers were identified that exhibited low μM IC_50_ values in fluorescence anisotropy competition assays and nM *K*_d_ as demonstrated in BLitzt (For - teBio) assays. Docking analyses suggested the Affimer, similarly to the phage derived peptides, bound p300 in the HIF-1α helix-3 binding cleft. The similar binding sites proposed for both the phage peptides and Affimers may suggest this is a crucial region for inhibitor design/targeting.

## Designed inhibitors of HIF PPIs – peptide, peptidomimetics and proteomimetics

**Fig. 8:**
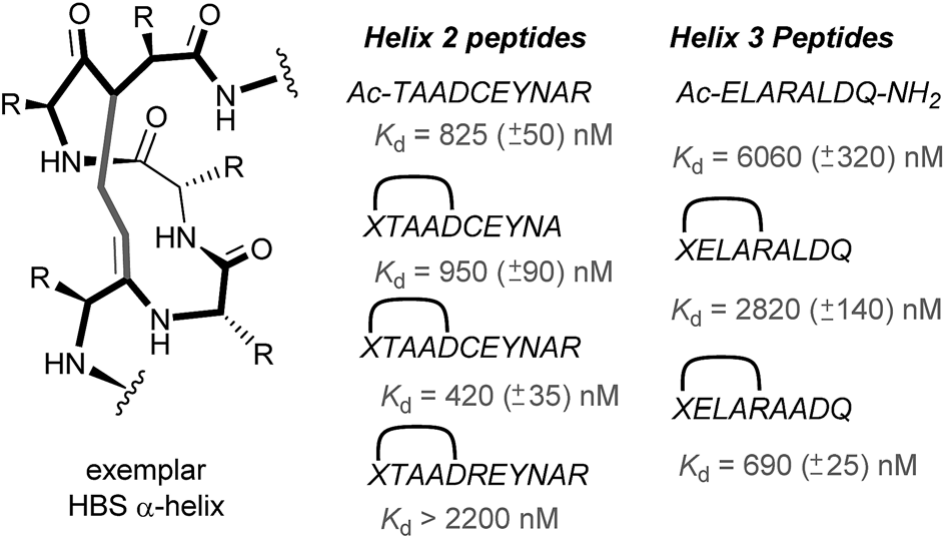
Hydrogen-bond surrogate constrained peptides that mimic helix 2 and 3 of HIF-1α as inhibitors of the HIF-1α/p300 interaction.

In 2010, work by the Arora group reduced the size of the HIF-1α/p300 interaction interface by focusing on one of the two key helices.^120^ An ITC binding experiment between the CH1 domain of p300 and C-TAD HIF-1α_799_Ac-TAADCEYNAR_804_ which corresponds to the helix 2 region; encompassing the binding residues Cys800 and Asn803 established this short peptide region had a binding affinity to p300 of 825 nM. Short peptides do not typically retain their folded conformation once excised from the protein environment. To stabilize this helical region Arora's team utilized the hydrogen bond surrogate (HBS) approach (Fig. 8).^121^ Three hydrogen bond surrogates (one negative control) were synthesized and CD used to show all 3 adopted a more helical conformation than the unconstrained peptide. The most potent ligand for p300 had a binding affinity of *K*_d_ = 420 nM as shown by ITC. The potential for the HBSs to down-regulate the HIF-1α induced transcription of VEGF gene in HeLa cells under hypoxic conditions was assessed by real-time quantitative polymerase chain reactions (qRT-PCR), the most potent HBS stabilized peptide showed a comparable level of transcriptional inhibition to chetomin (positive control). Further analysis indicated that the constrained peptide did not disrupt the interaction by denaturation of p300 in the same way as has been shown with chetomin whilst a cell viability assay demonstrated that the constrained peptide does not display significant cytotoxicity.

Subsequent work by the Arora group focused on the helix 3 region (Fig. 8).^69^ A computational alanine scan was conducted which suggested that Leu822, Asp823 and Gln824 were key binding residues and Leu819 less important. HBS peptides were prepared based on the sequence ELARALDQ, ensuring these three residues were retained (Leu822, Asp823 and Gln824): the constrained variant was expected to have the highest potency, whilst a constrained peptide bearing a point mutation to a key binding residue (Leu822–Ala822), was expected to bind with a lower affinity acting as a negative control and finally the unconstrained peptide was evaluated to permit the effect of helix stabilisation to be determined. The constrained peptides were shown to have enhanced helicity in comparison to the unconstrained peptide as shown by circular dichroism, whilst the affinity of the designed inhibitor as measured by tryptophan fluorescence spectroscopy, was impressive (*K*_d_ = 690 ± 25 nM), in comparison to the point mutant (negative control with point mutation, *K*_d_ = 2820 ± 140 nM) and the unconstrained analogue, *K*_d_ = 6060 ± 320 nM. Evidence for binding in the helix 3 binding pocket was obtained from HSQC NMR experiments, with prominent shifts occurring for residues around the helix 3 binding site, including Trp403, whilst inhibition of HIF-1α/p300 was demonstrated using a fluorescence polarisation competition assay (*K*_i_ = 1.2 μM). A luciferase-based reporter gene system was used to demonstrate down-regulation of hypoxia-inducible promoter activity *in cellulo* resulting in 25% reduced luciferase expression at 50 μM HBS-1. The ability of HBS-1 to inhibit hypoxia-induced transcription of target genes (VEGFA, SLC2A1/GLUT-1, and LOX) was evaluated using qRT-PCR assays. These demonstrated that HBS-1 reduced expression levels of these proteins in a dose dependent manner. HBS-1 was retained in plasma at much higher concentrations compared with the unconstrained peptide suggesting that the internally constrained structure of HBS-1 impacts favourably on serum stability and finally, a mouse xenograft tumour model was used to assess the *in vivo* efficacy of HBS-1 with promising results. Throughout the course of the treatment and at the experiment endpoint, mice treated with HBS 1 had smaller tumours with median tumour volume reduction of 53% compared with the mice from the control group.

**Fig. 9:**
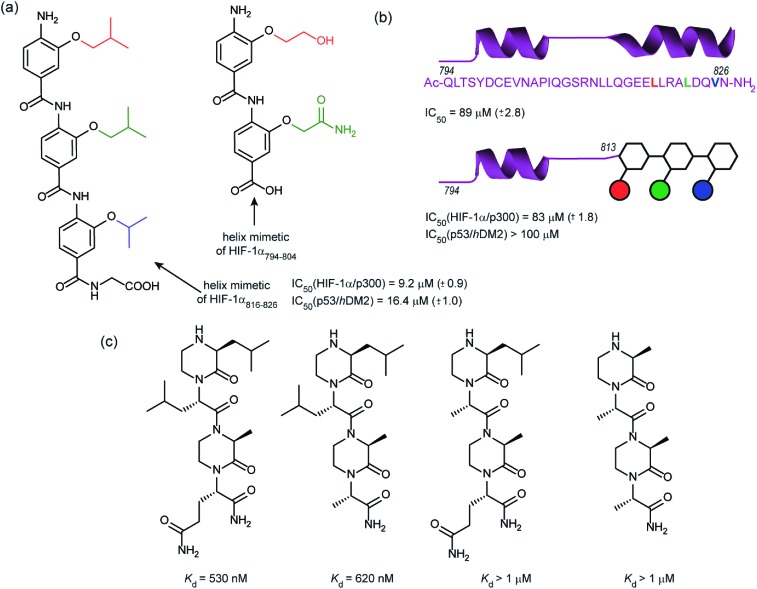
Helix mimetics as inhibitors of the HIF-1α/p300 interaction (a) aromatic oligoamide helix mimetics of helix 1 and 2 (b) “bionic” protein approach for HIF-1α/p300 interaction whereby a segment of HIF-1α is replaced with an aromatic oligoamide helix mimetic leading to comparable affinity to the peptide sequence and superior selectivity to the aromatic oligoamide helix mimetic for the hybrid (c) oxopiperazine helix mimetics of HIF-1α helix 3.

In 2014, our group employed a proteomimetic approach to identify inhibitors of the HIF-1α/p300 interaction, based on a trimeric 3-*O*-alkylated aromatic oligoamide (Fig. 9a).^108^ This scaffold has been designed to project the alkoxy group in such a manner as to reproduce the 3D spatial and angular projection of side chains from the *i*, *i* + 4 and *i* + 7 positions of a peptide adopting an α-helical conformation.^122^ The scaffold is amenable to solid-phase synthesis and had previously been used to construct p53/*h*DM2 inhibitors.^123,124^ In our first study helix 3 of HIF-1α was selected for mimicry; scaffolds with R1 = R2 = isobutyl, R3 = isopropyl (and the reverse sequence) were designed based on the previously annotated hot-spot residues. The best of these compounds was shown to act as a competitive inhibitor of HIF-1α/p300 in a fluorescence anisotropy assay (IC_50_ = 9.2 μM). A limited SAR study highlighted the need to have appropriate sides chains. Smaller and hydrophilic side-chains in any position abrogated binding as did the introduction of larger side chains (*e.g.* benzyl). The nature of the scaffold was also shown to be important: using an *N*-alkylated scaffold (also introduced previously by our group)^125^ functionalized with identical side chains, no inhibition was observed. Finally, the compound was shown to be selective over another helix mediated PPI; the eIF4E/eIF4G interaction.^126^ Subsequently our group applied this approach to the design of dimeric 3-*O*-alkylated aromatic oligoamide mimetics of the helix 2 sequence (Fig. 9a),^127^ however these compounds did not act as inhibitors in fluorescence competition anisotropy assays.

Although our original study indicated some selectivity for the HIF-1α/p300 interaction, selectivity was moderate, with some inhibition of p53/*h*DM2 being observed. To circumvent this deficiency we created hybrid structures comprising a peptide segment (from helix 2 of HIF-1α) and the original proteomimetic compound (mimicking helix 3 of HIF-1α) in an approach we referred to as a “bionic protein”.^128^ Although the potency of the hybrid was slightly diminished, it was comparable in potency to the helix_2–3_ peptide sequence upon which it was based and crucially the p53/*h*DM2 inhibition was diminished to a greater extent thus improving selectivity. This is also the first example of functionally indistinguishable incorporation of a topographical helix mimetic into a peptide sequence.

Simultaneously to our 2014 study, the Arora group reported on the use of an oxopiperazine helix mimetic (OHMs) scaffold to target the HIF-1α helix 3 binding site on p300.^70,129^ OHMs are assembled from naturally occurring amino acids with the nitrogen atoms of neighbouring backbone amides constrained with ethylene bridges providing a non-peptidic chiral scaffold that displays protein-like functionality as the bridges confine the side chain groups in orientations that mimic α-helices. Molecular modelling indicated that the low-energy conformation of the oxopiperazine scaffold presents side chain functionality to mimic the arrangement of the *i*, *i* + 4, and the *i* + 6/*i* + 7 residues on α-helices. Moreover, the chiral backbone of the oxopiperazine is expected to promote more effective and higher specificity binding to protein targets. Three of the key binding residues, Leu818, Leu822, and Gln824, were expected to be mimicked by oxopiperazine and four analogues were designed and synthesised to test this hypothesis (Fig. 9c), OHM-1 bears side chains representing all three key residues from HIF-1α: R1 as Leu818, R2 as Leu822, and R4 as Gln824. As the R3 position of the oxopiperazine scaffold was not predicted to make contacts with the target protein; an alanine residue was inserted at this position. OHMs 2 and 3 are single mutants of OHM-1 with R4 and R2 positions, respectively, substituted with alanine residues, whilst OHM-4 bears only alanine mimicking side chains. Intrinsic tryptophan fluorescence spectroscopy was used to measure binding affinity to the p300 CH1 domain. OHM-1, mimicking all three key side chains had the highest affinity of the OHMs whereas the remaining three mimetics all had lower affinity for p300 as expected. NMR was used to further characterise the binding; concentration-dependent shifts of several residues were observed upon addition of OHM-1, consistent with binding to the helix 3 region of p300. A luciferase-based reporter assay indicated dose-dependent reduction in the promoter activity, whilst the ability of OHMs to inhibit transcription of three selected HIF target genes, VEGFA, LOX, and GLUT1, was assessed using qRT-PCR assays in A549 cells. OHM1 at 10 μM down-regulated the mRNA expression levels of the critical angiogenesis regulator vascular endothelial growth factor (VEGFA) by 80%. In comparison, control compounds had no effect on VEGFA mRNA levels at these concentrations. Similar levels of decrease were observed for lysyl oxidase (LOX) and glucose transporter 1 (GLUT1) expression. Finally, the ability of OHM-1 to reduce the tumour growth rate in a mouse xenograft models was assessed; the treated group had a smaller median tumour volume (103 mm^3^) compared with the control group (186 mm^3^). This indicated that OMH-1, a mimetic of the helix 3 region of HIF-1α, is a potential cancer therapeutic.

## Conclusions

HIF represents an attractive and promising target in tumour metabolism. A number of successful approaches have been developed to modulate the supply of HIF upstream of its functional interactions, however these are regulated through protein–protein interactions (PPIs) making HIF a challenging target for molecular inhibition. Direct inhibition of HIF PPIs will allow the role of individual interactions within hypoxic signalling to be unpicked and may in the long term lead to bespoke cancer therapies. HIF PPIs are structurally more diverse, larger and complex than those PPI targets that have now become established as “ligandable” using small molecules (*e.g. h*DM2 and Bcl-2 family) as evidenced by the relatively high concentrations required for activity in many cases.

Crystal structures of key PPIs would facilitate chemical probe development as has been amply demonstrated for the HIF-2α PAS domain. Clearly, a crystal structure of the HIF-1α/p300 complex would allow additional efforts towards rational design but this complex has proven recalcitrant to crystallography, resisting significant efforts in both our laboratories and others. Our biological selection results appear to highlight a particular region of p300, confirmed by mutational analysis, which may prove important in future inhibitor design. Additionally, the recent work of Berlow *et al.* showing a CITED induced HIF-1 de-complexation from p300 (ref. 130) may lead to the identification of a potential allosteric site which weakens the interaction between HIF-1α and p300.

Despite these difficulties considerable progress has been made in harnessing both conventional drug discovery methodologies together with novel biological ligand selection tools and small molecule design strategies to identify inhibitors of a number of HIF PPIs (as shown in Table 2 and additional recent reviews).^131^ What is evident however, is that higher affinity ligands are required and with more desirable properties; indeed a number of those HIF-1 modulators discovered to date incorporate michael acceptors, fluorescent dyes *etc.* that would arouse concern amongst medicinal chemists particularly in the context of PAINS.^132^

**Table 2:** Selected examples of HIF modulators. Errors are given where available

Ligand	Target	Potency	Ref.
EZN-2698	mRNA	IC_50_ = 1–5 nM	75
Topotecan	Topoisomerase I	IC_50_ = 11 ± 1.3 μM	76
EZN-2208	Topoisomerase I	IC_50_ = 0.5 ± 0.3 μM	78
Digoxin	HIF-1α protein expression	IC_50_ = 50 nm	79
PX-478	HIF-1α protein expression	IC_50_ = 20 ± 2 μM	80
DMOG	PHD2	IC_50_ = 9.3 μM	82
FG-2216	PHD2	IC_50_ = 0.3 μM	85
Geldanamycin	HSP90	*K*_d_ = 1.21 μM	88
Ganetespib	HSP90	IC_50_ = 4 nM	90
Echinomycin	HRE	IC_50_ = 1.2 nM	96
Acriflavine	HIF-1α/β	IC_50_ = 1 μM	101
Chetomin	Zinc ejection	IC_50_ = 6.8 μM	32
Ninhydrin	Zinc ejection	IC_50_ = 1.93 ± 0.97 μM	104
KCN-1	HIF-1α/p300	IC_50_ = 0.65 ± 0.09 μM	105
KG548	ARNT/TACC3	IC_50_ = 25 μM	109
KHS101	ARNT	IC_50_ < 5 μM	109
cyclo-CLLFVY	HIF-1α/β	*K*_d_ = 124 ± 23 nM	112
Phage display peptides	p300	*K*_d_ = 20.67 ± 3.17 μM	65
Phage display Affimers	p300	*K*_d_ = 157 nM	65
HBS peptide helix 2	p300	*K*_d_ = 420 ± 35 nM	121
HBS peptide helix 3	p300	*K*_d_ = 690 ± 25 nM	74
Oligoamide 1	p300	IC_50_ = 9.2 ± 0.9 μM	108
OHM-1	p300	*K*_d_ = 420 nM	70 and 129

On a more positive note, the identification of HIF-2 allosteric inhibitors has validated this as a target in renal cancer, whilst HIF-1α/p300 helix mimetics have been shown to act in mouse tumour models. These examples highlight a promising future for further investigation in this area. We envision that as the field progresses, more potent and drug like compounds will become available for many of the other PPIs discussed above.

## References

[cit1] Hanahan D., Weinberg R. A. (2011). Cell.

[cit2] Manalo D. J., Rowan A., Lavoie T., Natarajan L., Kelly B. D., Ye S. Q., Garcia J. G. N., Semenza G. L. (2005). Blood.

[cit3] Vaupel P., Thews O., Kelleher D. K. and Hoeckel M., in Oxygen Transport to Tissue XX, ed. A. G. Hudetz and D. F. Bruley, Springer US, Boston, MA, 1998, pp. 591–602, 10.1007/978-1-4615-4863-8_70.

[cit4] Semenza G. L. (2003). Nat. Rev. Cancer.

[cit5] Semenza G. L. (2002). Biochem. Pharmacol..

[cit6] Pugh C. W., Ratcliffe P. J. (2003). Nat. Med..

[cit7] Huang L. E., Bunn H. F. (2003). J. Biol. Chem..

[cit8] Harris A. L. (2002). Nat. Rev. Cancer.

[cit9] Nordgren I. K., Tavassoli A. (2011). Chem. Soc. Rev..

[cit10] Semenza G. L. (2011). N. Engl. J. Med..

[cit11] Zhong H., De Marzo A. M., Laughner E., Lim M., Hilton D. A., Zagzag D., Buechler P., Isaacs W. B., Semenza G. L., Simons J. W. (1999). Cancer Res..

[cit12] Semenza G. L. (2009). Oncogene.

[cit13] Höckel M., Vaupel P. (2001). Semin. Oncol..

[cit14] Selak M. A., Armour S. M., MacKenzie E. D., Boulahbel H., Watson D. G., Mansfield K. D., Pan Y., Simon M. C., Thompson C. B., Gottlieb E. (2005). Cancer Cell.

[cit15] Dales J.-P., Garcia S., Meunier-Carpentier S., Andrac-Meyer L., Haddad O., Lavaut M.-N., Allasia C., Bonnier P., Charpin C. (2005). Int. J. Cancer.

[cit16] Semenza G. L. (2000). Crit. Rev. Biochem. Mol. Biol..

[cit17] Ivan M., Kondo K., Yang H., Kim W., Valiando J., Ohh M., Salic A., Asara J. M., Lane W. S., Kaelin Jr W. G. (2001). Science.

[cit18] Jaakkola P., Mole D. R., Tian Y. M., Wilson M. I., Gielbert J., Gaskell S. J., von Kriegsheim A., Hebestreit H. F., Mukherji M., Schofield C. J., Maxwell P. H., Pugh C. W., Ratcliffe P. J. (2001). Science.

[cit19] Powis G., Kirkpatrick L. (2004). Mol. Cancer Ther..

[cit20] Ravi R., Mookerjee B., Bhujwalla Z. M., Sutter C. H., Artemov D., Zeng Q., Dillehay L. E., Madan A., Semenza G. L., Bedi A. (2000). Genes Dev..

[cit21] Gradin K., McGuire J., Wenger R. H., Kvietikova I., fhitelaw M. L., Toftgård R., Tora L., Gassmann M., Poellinger L. (1996). Mol. Cell. Biol..

[cit22] Arany Z., Huang L. E., Eckner R., Bhattacharya S., Jiang C., Goldberg M. A., Bunn H. F., Livingston D. M. (1996). Proc. Natl. Acad. Sci. U. S. A..

[cit23] Wang F., Marshall C., Ikura M. (2013). Cell. Mol. Life Sci..

[cit24] Lando D., Peet D. J., Whelan D. A., Gorman J. J., Whitelaw M. L. (2002). Science.

[cit25] Chowdhury R., Hardy A., Schofield C. J. (2008). Chem. Soc. Rev..

[cit26] Liao D., Johnson R. S. (2007). Cancer Metastasis Rev..

[cit27] Wang Y., Liu Y., Malek S. N., Zheng P., Liu Y. (2011). Cell Stem Cell.

[cit28] Zhang H., Gao P., Fukuda R., Kumar G., Krishnamachary B., Zeller K. I., Dang C. V., Semenza G. L. (2007). Cancer Cell.

[cit29] Franovic A., Gunaratnam L., Smith K., Robert I., Patten D., Lee S. (2007). Proc. Natl. Acad. Sci. U. S. A..

[cit30] Erler J. T., Bennewith K. L., Nicolau M., Dornhofer N., Kong C., Le Q.-T., Chi J.-T. A., Jeffrey S. S., Giaccia A. J. (2006). Nature.

[cit31] Semenza G. L. (2012). Trends Pharmacol. Sci..

[cit32] Kung A. L., Zabludoff S. D., France D. S., Freedman S. J., Tanner E. A., Vieira A., Cornell-Kennon S., Lee J., Wang B., Wang J., Memmert K., Naegeli H.-U., Petersen F., Eck M. J., Bair K. W., Wood A. W., Livingston D. M. (2004). Cancer Cell.

[cit33] Cardoso R., Love R., Nilsson C. L., Bergqvist S., Nowlin D., Yan J., Liu K. K. C., Zhu J., Chen P., Deng Y.-L., Dyson H. J., Greig M. J., Brooun A. (2012). Protein Sci..

[cit34] Guo Y., Scheuermann T. H., Partch C. L., Tomchick D. R., Gardner K. H. (2015). J. Biol. Chem..

[cit35] Scheuermann T. H., Tomchick D. R., Machius M., Guo Y., Bruick R. K., Gardner K. H. (2009). Proc. Natl. Acad. Sci. U. S. A..

[cit36] Rogers J. L., Bayeh L., Scheuermann T. H., Longgood J., Key J., Naidoo J., Melito L., Shokri C., Frantz D. E., Bruick R. K., Gardner K. H., MacMillan J. B., Tambar U. K. (2013). J. Med. Chem..

[cit37] Scheuermann T. H., Li Q., Ma H.-W., Key J., Zhang L., Chen R., Garcia J. A., Naidoo J., Longgood J., Frantz D. E., Tambar U. K., Gardner K. H., Bruick R. K. (2013). Nat. Chem. Biol..

[cit38] Cho H., Du X., Rizzi J. P., Liberzon E., Chakraborty A. A., Gao W., Carvo I., Signoretti S., Bruick R. K., Josey J. A., Wallace E. M., Kaelin W. G. (2016). Nature.

[cit39] Wallace E. M., Rizzi J. P., Han G., Wehn P. M., Cao Z., Du X., Cheng T., Czerwinski R. M., Dixon D. D., Goggin B. S., Grina J. A., Halfmann M. M., Maddie M. A., Olive S. R., Schlachter S. T., Tan H., Wang B., Wang K., Xie S., Xu R., Yang H., Josey J. A. (2016). Cancer Res..

[cit40] Scheuermann T. H., Stroud D., Sleet C. E., Bayeh L., Shokri C., Wang H., Caldwell C. G., Longgood J., MacMillan J. B., Bruick R. K., Gardner K. H., Tambar U. K. (2015). J. Med. Chem..

[cit41] Key J., Scheuermann T. H., Anderson P. C., Daggett V., Gardner K. H. (2009). J. Am. Chem. Soc..

[cit42] Elkins J. M., Hewitson K. S., McNeill L. A., Seibel J. F., Schlemminger I., Pugh C. W., Ratcliffe P. J., Schofield C. J. (2003). J. Biol. Chem..

[cit43] Hangasky J. A., Taabazuing C. Y., Martin C. B., Eron S. J., Knapp M. J. (2017). J. Inorg. Biochem..

[cit44] Hewitson K. S., Holmes S. L., Ehrismann D., Hardy A. P., Chowdhury R., Schofield C. J., McDonough M. A. (2008). J. Biol. Chem..

[cit45] Chowdhury R., Leung I. K. H., Tian Y.-M., Abboud M. I., Ge W., Domene C., Cantrelle F.-X., Landrieu I., Hardy A. P., Pugh C. W., Ratcliffe P. J., Claridge T. D. W., Schofield C. J. (2016). Nat. Commun..

[cit46] Van Molle I., Thomann A., Buckley D. L., So E. C., Lang S., Crews C. M., Ciulli A. (2012). Chem. Biol..

[cit47] Hon W.-C., Wilson M. I., Harlos K., Claridge T. D. W., Schofield C. J., Pugh C. W., Maxwell P. H., Ratcliffe P. J., Stuart D. I., Jones E. Y. (2002). Nature.

[cit48] Min J.-H., Yang H., Ivan M., Gertler F., Kaelin W. G., Pavletich N. P. (2002). Science.

[cit49] Jiang B.-H., Rue E., Wang G. L., Roe R., Semenza G. L. (1996). J. Biol. Chem..

[cit50] Li H., Ko H. P., Whitlock J. P. (1996). J. Biol. Chem..

[cit51] Tanaka T., Wiesener M., Bernhardt W., Eckardt K.-U., Warnecke C. (2009). Biochem. J..

[cit52] Wiesener M. S., Jürgensen J. S., Rosenberger C., Scholze C. K., Hörstrup J. H., Warnecke C., Mandriota S., Bechmann I., Frei U. A., Pugh C. W., Ratcliffe P. J., Bachmann S., Maxwell P. H., Eckardt K.-U. (2003). FASEB J..

[cit53] Jürgensen J. S., Rosenberger C., Wiesener M. S., Warnecke C., Hörstrup J. H., Gräfe M., Philipp S., Griethe W., Maxwell P. H., Frei U., Bachmann S., Willenbrock R., Eckardt K.-U. (2004). FASEB J..

[cit54] Wang G. L., Jiang B. H., Rue E. A., Semenza G. L. (1995). Proc. Natl. Acad. Sci. U. S. A..

[cit55] Wu D., Potluri N., Lu J., Kim Y., Rastinejad F. (2015). Nature.

[cit56] Huang Z. J., Edery I., Rosbash M. (1993). Nature.

[cit57] Goodman R. H., Smolik S. (2000). Genes Dev..

[cit58] Vo N., Goodman R. H. (2001). J. Biol. Chem..

[cit59] Kallio P. J., Okamoto K., O'Brien S., Carrero P., Makino Y., Tanaka H., Poellinger L. (1998). EMBO J..

[cit60] Bragança J., Swingler T., Marques F. I. R., Jones T., Eloranta J. J., Hurst H. C., Shioda T., Bhattacharya S. (2002). J. Biol. Chem..

[cit61] Bhattacharya S., Michels C. L., Leung M.-K., Arany Z. P., Kung A. L., Livingston D. M. (1999). Genes Dev..

[cit62] Freedman S. J., Sun Z.-Y. J., Poy F., Kung A. L., Livingston D. M., Wagner G., Eck M. J. (2002). Proc. Natl. Acad. Sci. U. S. A..

[cit63] Dames S. A., Martinez-Yamout M., De Guzman R. N., Dyson H. J., Wright P. E. (2002). Proc. Natl. Acad. Sci. U. S. A..

[cit64] Ruas J. L., Berchner-Pfannschmidt U., Malik S., Gradin K., Fandrey J., Roeder R. G., Pereira T., Poellinger L. (2010). J. Biol. Chem..

[cit65] Kyle H. F., Wickson K. F., Stott J., Burslem G. M., Breeze A. L., Tiede C., Tomlinson D. C., Warriner S. L., Nelson A., Wilson A. J., Edwards T. A. (2015). Mol. BioSyst..

[cit66] Cho H., Ahn D.-R., Park H., Yang E. G. (2007). FEBS Lett..

[cit67] Gu J., Milligan J., Huang L. E. (2001). J. Biol. Chem..

[cit68] Ruas J. L., Poellinger L., Pereira T. (2002). J. Biol. Chem..

[cit69] Kushal S., Lao B. B., Henchey L. K., Dubey R., Mesallati H., Traaseth N. J., Olenyuk B. Z., Arora P. S. (2013). Proc. Natl. Acad. Sci. U. S. A..

[cit70] Lao B. B., Grishagin I., Mesallati H., Brewer T. F., Olenyuk B. Z., Arora P. S. (2014). Proc. Natl. Acad. Sci. U. S. A..

[cit71] Beischlag T. V., Taylor R. T., Rose D. W., Yoon D., Chen Y., Lee W.-H., Rosenfeld M. G., Hankinson O. (2004). J. Biol. Chem..

[cit72] Kim J. H., Stallcup M. R. (2004). J. Biol. Chem..

[cit73] Partch C. L., Gardner K. H. (2011). Proc. Natl. Acad. Sci. U. S. A..

[cit74] Young R. M., Wang S.-J., Gordan J. D., Ji X., Liebhaber S. A., Simon M. C. (2008). J. Biol. Chem..

[cit75] Greenberger L. M., Horak I. D., Filpula D., Sapra P., Westergaard M., Frydenlund H. F., Albæk C., Schrøder H., Ørum H. (2008). Mol. Cancer Ther..

[cit76] Beppu K., Nakamura K., Linehan W. M., Rapisarda A., Thiele C. J. (2005). Cancer Res..

[cit77] Rapisarda A., Hollingshead M., Uranchimeg B., Bonomi C. A., Borgel S. D., Carter J. P., Gehrs B., Raffeld M., Kinders R. J., Parchment R., Anver M. R., Shoemaker R. H., Melillo G. (2009). Mol. Cancer Ther..

[cit78] Sapra P., Zhao H., Mehlig M., Malaby J., Kraft P., Longley C., Greenberger L. M., Horak I. D. (2008). Clin. Cancer Res..

[cit79] Zhang H., Qian D. Z., Tan Y. S., Lee K., Gao P., Ren Y. R., Rey S., Hammers H., Chang D., Pili R., Dang C. V., Liu J. O., Semenza G. L. (2008). Proc. Natl. Acad. Sci. U. S. A..

[cit80] Koh M. Y., Spivak-Kroizman T., Venturini S., Welsh S., Williams R. R., Kirkpatrick D. L., Powis G. (2008). Mol. Cancer Ther..

[cit81] Chan M. C., Holt-Martyn J. P., Schofield C. J., Ratcliffe P. J. (2016). Mol. Aspects Med..

[cit82] Epstein A. C. R., Gleadle J. M., McNeill L. A., Hewitson K. S., O'Rourke J., Mole D. R., Mukherji M., Metzen E., Wilson M. I., Dhanda A., Tian Y.-M., Masson N., Hamilton D. L., Jaakkola P., Barstead R., Hodgkin J., Maxwell P. H., Pugh C. W., Schofield C. J., Ratcliffe P. J. (2001). Cell.

[cit83] Ivan M., Haberberger T., Gervasi D. C., Michelson K. S., Günzler V., Kondo K., Yang H., Sorokina I., Conaway R. C., Conaway J. W., Kaelin W. G. (2002). Proc. Natl. Acad. Sci. U. S. A..

[cit84] McDonough M. A., McNeill L. A., Tilliet M., Papamicaël C. A., Chen Q.-Y., Banerji B., Hewitson K. S., Schofield C. J. (2005). J. Am. Chem. Soc..

[cit85] Chowdhury R., Candela-Lena J. I., Chan M. C., Greenald D. J., Yeoh K. K., Tian Y.-M., McDonough M. A., Tumber A., Rose N. R., Conejo-Garcia A., Demetriades M., Mathavan S., Kawamura A., Lee M. K., van Eeden F., Pugh C. W., Ratcliffe P. J., Schofield C. J. (2013). ACS Chem. Biol..

[cit86] Provenzano R., Besarab A., Sun C. H., Diamond S. A., Durham J. H., Cangiano J. L., Aiello J. R., Novak J. E., Lee T., Leong R., Roberts B. K., Saikali K. G., Hemmerich S., Szczech L. A., Yu K.-H. P., Neff T. B. (2016). Clin. J. Am. Soc. Nephrol..

[cit87] Taipale M., Jarosz D. F., Lindquist S. (2010). Nat. Rev. Mol. Cell Biol..

[cit88] Li H., Sedgwick A. C., Li M., Blackburn R. A. R., Bull S. D., Arbault S., James T. D., Sojic N. (2016). Chem. Commun..

[cit89] Yang C., Wang W., Chen L., Liang J., Lin S., Lee M.-Y., Ma D.-L., Leung C.-H. (2016). Chem. Commun..

[cit90] Xiang L., Gilkes D. M., Chaturvedi P., Luo W., Hu H., Takano N., Liang H., Semenza G. L. (2014). J. Mol. Med..

[cit91] Buckley D. L., Van Molle I., Gareiss P. C., Tae H. S., Michel J., Noblin D. J., Jorgensen W. L., Ciulli A., Crews C. M. (2012). J. Am. Chem. Soc..

[cit92] Buckley D. L., Gustafson J. L., Van Molle I., Roth A. G., Tae H. S., Gareiss P. C., Jorgensen W. L., Ciulli A., Crews C. M. (2012). Angew. Chem., Int. Ed..

[cit93] Galdeano C., Gadd M. S., Soares P., Scaffidi S., Van Molle I., Birced I., Hewitt S., Dias D. M., Ciulli A. (2014). J. Med. Chem..

[cit94] Frost J., Galdeano C., Soares P., Gadd M. S., Grzes K. M., Ellis L., Epemolu O., Shimamura S., Bantscheff M., Grandi P., Read K. D., Cantrell D. A., Rocha S., Ciulli A. (2016). Nat. Commun..

[cit95] Toure M., Crews C. M. (2016). Angew. Chem., Int. Ed. Engl..

[cit96] Kong D., Park E. J., Stephen A. G., Calvani M., Cardellina J. H., Monks A., Fisher R. J., Shoemaker R. H., Melillo G. (2005). Cancer Res..

[cit97] Nickols N. G., Jacobs C. S., Farkas M. E., Dervan P. B. (2007). ACS Chem. Biol..

[cit98] Dervan P. B., Edelson B. S. (2003). Curr. Opin. Struct. Biol..

[cit99] Concellón A., Blasco E., Martínez-Felipe A., Martínez J. C., Šics I., Ezquerra T. A., Nogales A., Piñol M., Oriol L. (2016). Macromolecules.

[cit100] Shin D. H., Chun Y.-S., Lee D. S., Huang L. E., Park J.-W. (2008). Blood.

[cit101] Lee K., Zhang H., Qian D. Z., Rey S., Liu J. O., Semenza G. L. (2009). Proc. Natl. Acad. Sci. U. S. A..

[cit102] Cook K. M., Hilton S. T., Mecinovic J., Motherwell W. B., Figg W. D., Schofield C. J. (2009). J. Biol. Chem..

[cit103] Dubey R., Levin M. D., Szabo L. Z., Laszlo C. F., Kushal S., Singh J. B., Oh P., Schnitzer J. E., Olenyuk B. Z. (2013). J. Am. Chem. Soc..

[cit104] Jayatunga M. K. P., Thompson S., McKee T. C., Chan M. C., Reece K. M., Hardy A. P., Sekirnik R., Seden P. T., Cook K. M., McMahon J. B., Figg W. D., Schofield C. J., Hamilton A. D. (2015). Eur. J. Med. Chem..

[cit105] Tan C., de Noronha R. G., Devi N. S., Jabbar A. A., Kaluz S., Liu Y., Mooring S. R., Nicolaou K. C., Wang B., Van Meir E. G. (2011). Bioorg. Med. Chem. Lett..

[cit106] Mooring S. R., Jin H., Devi N. S., Jabbar A. A., Kaluz S., Liu Y., Van Meir E. G., Wang B. (2011). J. Med. Chem..

[cit107] Yin S., Kaluz S., Devi N. S., Jabbar A. A., de Noronha R. G., Mun J., Zhang Z., Boreddy P. R., Wang W., Wang Z., Abbruscato T., Chen Z., Olson J. J., Zhang R., Goodman M. M., Nicolaou K. C., Van Meir E. G. (2012). Clin. Cancer Res..

[cit108] Burslem G. M., Kyle H. F., Breeze A. L., Edwards T. A., Nelson A., Warriner S. L., Wilson A. J. (2014). ChemBioChem.

[cit109] Guo Y., Partch C. L., Key J., Card P. B., Pashkov V., Patel A., Bruick R. K., Wurdak H., Gardner K. H. (2012). ACS Chem. Biol..

[cit110] Chen W., Hill H., Christie A., Kim M.
S., Holloman E., Pavia-Jimenez A., Homayoun F., Ma Y., Patel N., Yell P., Hao G., Yousuf Q., Joyce A., Pedrosa I., Geiger H., Zhang H., Chang J., Gardner K. H., Bruick R. K., Reeves C., Hwang T. H., Courtney K., Frenkel E., Sun X., Zojwalla N., Wong T., Rizzi J. P., Wallace E. M., Josey J. A., Xie Y., Xie X.-J., Kapur P., McKay R. M., Brugarolas J. (2016). Nature.

[cit111] Tavassoli A., Benkovic S. J. (2007). Nat. Protoc..

[cit112] Miranda E., Nordgren I. K., Male A. L., Lawrence C. E., Hoakwie F., Cuda F., Court W., Fox K. R., Townsend P. A., Packham G. K., Eccles S. A., Tavassoli A. (2013). J. Am. Chem. Soc..

[cit113] Mistry I. N., Tavassoli A. (2017). ACS Synth. Biol..

[cit114] Williamson D. J., Webb M. E., Turnbull W. B. (2014). Nat. Protoc..

[cit115] Devlin J., Panganiban L., Devlin P. (1990). Science.

[cit116] Cwirla S. E., Peters E. A., Barrett R. W., Dower W. J. (1990). Proc. Natl. Acad. Sci. U. S. A..

[cit117] Scott J., Smith G. (1990). Science.

[cit118] Matochko W. L., Chu K., Jin B., Lee S. W., Whitesides G. M., Derda R. (2012). Methods.

[cit119] Tiede C., Tang A. A. S., Deacon S. E., Mandal U., Nettleship J. E., Owen R. L., George S. E., Harrison D. J., Owens R. J., Tomlinson D. C., McPherson M. J. (2014). Protein Eng., Des. Sel..

[cit120] Henchey L. K., Kushal S., Dubey R., Chapman R. N., Olenyuk B. Z., Arora P. S. (2010). J. Am. Chem. Soc..

[cit121] Patgiri A., Jochim A. L., Arora P. S. (2008). Acc. Chem. Res..

[cit122] Burslem G. M., Wilson A. J. (2014). Synlett.

[cit123] Azzarito V., Prabhakaran P., Bartlett A. I., Murphy N. S., Hardie M. J., Kilner C. A., Edwards T. A., Warriner S. L., Wilson A. J. (2012). Org. Biomol. Chem..

[cit124] Murphy N., Prabhakaran P., Azzarito V., Plante J., Hardie M., Kilner C., Warriner S., Wilson A. (2013). Chem.–Eur. J..

[cit125] Campbell F., Plante J. P., Edwards T. A., Warriner S. L., Wilson A. J. (2010). Org. Biomol. Chem..

[cit126] Brown C. J., Lim J. J., Leonard T., Lim H. C. A., Chia C. S. B., Verma C. S., Lane D. P. (2011). J. Mol. Biol..

[cit127] Burslem G. M., Kyle H. F., Prabhakaran P., Breeze A. L., Edwards T. A., Warriner S. L., Nelson A., Wilson A. J. (2016). Org. Biomol. Chem..

[cit128] Burslem G. M., Kyle H. F., Breeze A. L., Edwards T. A., Nelson A., Warriner S. L., Wilson A. J. (2016). Chem. Commun..

[cit129] Lao B. B., Drew K., Guarracino D. A., Brewer T. F., Heindel D. W., Bonneau R., Arora P. S. (2014). J. Am. Chem. Soc..

[cit130] Berlow R. B., Dyson H. J., Wright P. E. (2017). Nature.

[cit131] Martin A. R., Ronco C., Demange L., Benhida R. (2017). MedChemComm.

[cit132] Baell J., Walters M. A. (2014). Nature.

[cit133] Chowdhury R., McDonough M. A., Mecinovic J., Loenarz C., Flashman E., Hewitson K. S., Comene C. (2009). Structure.

