# Further Correction: Hypoxia inducible factor (HIF) as a model for studying inhibition of protein–protein interactions

**DOI:** 10.1039/c8sc90023j

**Published:** 2018-02-01

**Authors:** George M. Burslem, Hannah F. Kyle, Adam Nelson, Thomas A. Edwards, Andrew J. Wilson

**Affiliations:** a School of Chemistry , University of Leeds , Woodhouse Lane , Leeds LS2 9JT , UK . Email: a.j.wilson@leeds.ac.uk; b Astbury Centre for Structural Molecular Biology , University of Leeds , Woodhouse Lane , Leeds LS2 9JT , UK; c School of Molecular and Cellular Biology , Faculty of Biological Sciences , University of Leeds , Woodhouse Lane , Leeds LS2 9JT , UK

## Abstract

Correction for ‘Hypoxia inducible factor (HIF) as a model for studying inhibition of protein–protein interactions’ by George M. Burslem *et al.*, *Chem. Sci.*, 2017, **8**, 4188–4202.



## 

The authors regret that there are additional errors in the original manuscript that were not noted in the first correction notice.

Table 1 is incorrect in the original manuscript as a number of citations were incorrectly numbered and some PDB IDs were omitted from the original table. The correct table is displayed below.

**Table 1:** Summary of the currently available HIF structures

Structure	Structure details	PDB ID	Ref.
HIF dimers	HIF1α/ARNT	4H6J	33
	HIF2α/ARNT/co-activator complex	4PKY	34
	HIF2α/ARNT complex	3F1P	35
	HIF2α/ARNT complex with an artificial ligand bound	3F1O	
	HIF2α/ARNT complex	4ZP4	55
	HIF2α/ARNT complex with a benzoxadiazole ligand bound	4GS9, ; 4GHI	36 and 37
	HIF2α-ARNT bound to PT2399	5UFP	38
	HIF2α-ARNT bound to PT2385	5TBM	39
	HIF2α-ARNT PAS domain bound to tetrazole containing antagonist	4XT2	40
	HIF2α-ARNT complex bound to proflavin	4ZPH	55
	HIF2α-ARNT complex with HRE DNA	4ZPK	55
	HIF2α-ARNT bound to benzoxadiazole antagonist	4ZQD	55
	HIF1α-ARNT complex with HRE DNA	4ZPR	55
	HIF2α-ARNT bound to benzoxadiazole antagonist	4ZQD	55
	HIF2α-ARNT bound to THS017/THS020	3H7W, ; 3H82	41
	HIF2α-ARNT complex with ethylene glycol	3F1N	35
HIF-FIH complexes	FIH in complex with HIF-1α	1H2K, ; 1H2L, ; 1H2M	42
	FIH (D201E) complex with HIF-1α and α-ketoglutarate	5JWP	43
		3D8C, ; 2ILM	44
HIF-PHD complexes	PHD2 in complex with 2OG and HIF-1α CODD	5L9B, ; 5L9V, ; 5LA9	45
		5LAS	
	PHD2 in complex with NOG and HIF-1α	3HQR	133
vHL-HIF complexes	vHL/elongin/B-elongin/C-elongin complex bound to HIF-1α	4AJY	46
		1LQB	47
		1LM8	48
HIF-1α complexes	NMR structures of HIF-1a bound to p300	1L3E	62
		1L8C	63

Ref. 88 is incorrect in the original manuscript the correct citation is: J. S. Isaacs, Y.-J. Jung, E. G. Mimnaugh, A. Martinez, F. Cuttitta and L. M. Neckers, *J. Biol. Chem.*, 2002, **277**, 29936–29944.

Ref. 89 is incorrect in the original manuscript the correct citation is: E. Hur, H.-H. Kim, S. M. Choi, J. H. Kim, S. Yim, H. J. Kwon, Y. Choi, D. K. Kim, M.-O. Lee and H. Park, *Mol. Pharmacol.*, 2002, **62**, 975–982.

Ref. 99 is incorrect in the original manuscript the correct citation is: S. Kaluz, M. Kaluzová and E. J. Stanbridge, *Mol. Cell. Biol.*, 2006, **26**, 5895–5907.

Table 2 is also incorrect as a number of citations were incorrectly numbered. The correct table is displayed below.

**Table 2:** Selected examples of HIF modulators. Error are given where available

Ligand	Target	Potency	Ref.
EZN-2698	mRNA	IC_50_ = 1–5 nM	75
Topotecan	Topoisomerase I	IC_50_ = 11 ± 1.3 μM	76
EZN-2208	Topoisomerase I	IC_50_ = 0.5 ± 0.3 μM	78
Digoxin	HIF-1α protein expression	IC_50_ = 50 nM	79
PX-478	HIF-1α protein expression	IC_50_ = 20 ± 2 μM	80
DMOG	PHD2	IC_50_ = 9.3 μM	82
FG-2216	PHD2	IC_50_ = 0.3 μM	85
Geldanamycin	HSP90	*K*_d_ = 1.21 μM	88
Ganete spib	HSP90	IC_50_ = 4 nM	90
Echinomycin	HRE	IC_50_ = 1.2 nM	96
Acriflavine	HIF-1α/β	IC_50_ = 1 μM	101
Chetomin	Zinc ejection	IC_50_ = 6.8 μM	32
Ninhydrin	Zinc ejection	IC_50_ = 1.93 ± 0.97 μM	104
KCN-1	HIF-1α/p300	IC_50_ = 0.65 ± 0.09 μM	105
KG548	ARNT/TACC3	IC_50_ = 25 μM	109
KHS101	ARNT	IC_50_ < 5 μM	109
cyclo-CLLFVY	HIF-1α/β	*K*_d_ = 124 (± 23) nM	112
Phage display peptides	p300	*K*_d_ = 20.67 (± 23) μM	65
Phage display Affimers	p300	*K*_d_ = 157 nM	65
HBS peptide helix 2	p300	*K*_d_ = 420 nM	120
HBS peptide helix 3	p300	*K*_d_ = 690 ± 25 nM	69
Oligoamide 1	p300	IC_50_ = 9.2 μM	108
OHM-1	p300	*K*_d_ = 420 nM	70 and 129

Ref. 133 is incomplete in the original manuscript. The correct citation should be: R. Chowdhury, M. A. McDonough, J. Mecinović, C. Loenarz, E. Flashman, K. S. Hewitson, C. Domene and C. J. Schofield, *Structure*, 2009, **17**, 981–989.

The Royal Society of Chemistry apologises for these errors and any consequent inconvenience to authors and readers.

