# Correction: Correction: Hypoxia inducible factor (HIF) as a model for studying inhibition of protein–protein interactions

**DOI:** 10.1039/c8sc90024h

**Published:** 2018-02-02

**Authors:** George M. Burslem, Hannah F. Kyle, Adam Nelson, Thomas A. Edwards, Andrew J. Wilson

**Affiliations:** a School of Chemistry , University of Leeds , Woodhouse Lane , Leeds LS2 9JT , UK . Email: a.j.wilson@leeds.ac.uk; b Astbury Centre for Structural Molecular Biology , University of Leeds , Woodhouse Lane , Leeds LS2 9JT , UK; c School of Molecular and Cellular Biology , Faculty of Biological Sciences , University of Leeds , Woodhouse Lane , Leeds LS2 9JT , UK

## Abstract

Correction for ‘Correction: Hypoxia inducible factor (HIF) as a model for studying inhibition of protein–protein interactions’ by George M. Burslem *et al.*, *Chem. Sci.*, 2017, **8**, 5214–5215.



## 

The authors regret that in their original correction regarding Fig. 3 there remained errors in the structures of EZN-2208, geldanamycin, ganetespib and echinomycin. The correct figure is displayed below.

**Fig. 3:**
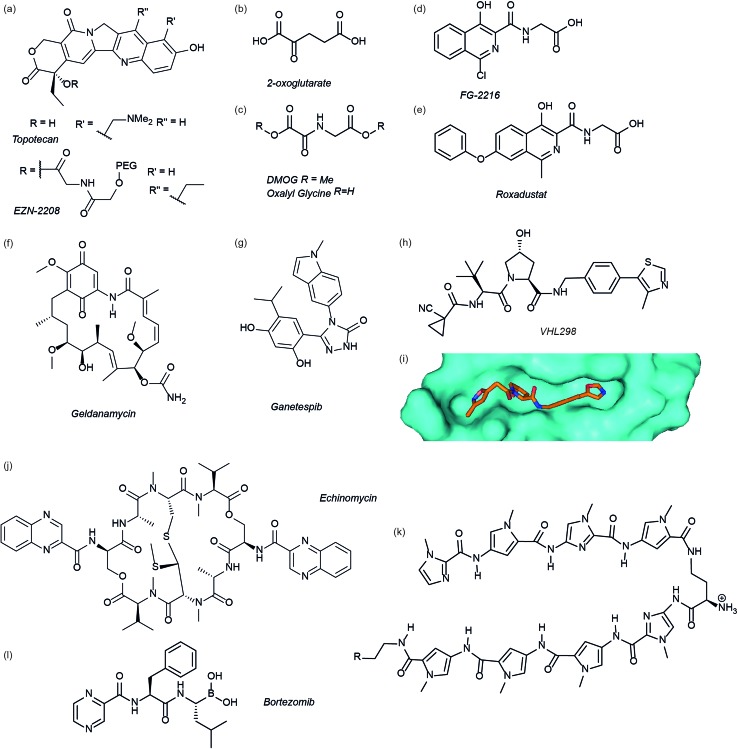
Modulators of the HIF pathway (a) topotecan and derivative EZN-2208 (b) 2-oxoglutarate (c) DMOG and oxalyl glycine (d) FG-2216 (e) roxadustat (f) geldanamycin (g) ganetespib (h) X-ray crystal structure of a hydroxyproline derived inhibitor (orange) bound to pVHL (cyan), PDB ID 3zrc (i) optimised hydroxyproline derived pVHL inhibitor VH298 (j) echinomycin (k) DNA sequence specific polyamide (l) bortezomib.

The Royal Society of Chemistry apologises for these errors and any consequent inconvenience to authors and readers.

